# How psychiatric trainees keep up to date: survey of psychiatric trainees' use of journals and other information sources

**DOI:** 10.1192/pb.bp.113.045682

**Published:** 2016-02

**Authors:** Tom Walker-Tilley, John Bainton, Matthew Fernando, Yimlun Wong, Ba Ko, James Warner, Ramin Nilforooshan

**Affiliations:** 1 South London and Maudsley NHS Foundation Trust; 2 Central and North West London NHS Foundation Trust; 3 Surrey and Borders Partnership NHS Foundation Trust

## Abstract

**Aims and method** To gather information about psychiatric trainees' use of different information sources and academic materials, a questionnaire was distributed at the London Deanery Annual Psychiatry Trainee Conference and the training programmes of two teaching trusts.

**Results** Participants returned 202 out of a total of 300 completed questionnaires (67%). Websites were the most commonly accessed information source ahead of textbooks, abstracts and journals. Year of training correlated positively with journal use and negatively with textbook use. Year of training also correlated positively with frequency of reading three journals published by the Royal College of Psychiatrists and with specific reasons for consulting journals, namely to improve clinical practice and inform trainees' own research.

**Clinical implications** Respondents reported consulting websites more frequently than more traditional information sources but journals are still a widely used source of information for trainee clinicians. It is important that trainees continue to be equipped with skills to identify and access high-quality information at the point of clinical uncertainty.

Publication of articles in peer-reviewed journals plays an important part in disseminating research findings and advances in practice to clinicians. The reading of such articles is one way in which doctors maintain their General Medical Council (GMC)-mandated duty to keep their knowledge and skills up to date.^1^ The importance of journals in educating psychiatric trainees is recognised by the Royal College of Psychiatrists. At core trainee level journal clubs form an assessed part of the curriculum and critical appraisal of published research evidence is assessed in the exams for College membership (MRCPsych).^2^ At higher trainee level, self-directed learning from peer-reviewed journals as well as library facilities that ‘include a minimum of 10 psychiatric journals as well as providing access to general medical journals’ is mandatory.^3^ However, academic journals are only one of many information sources available to trainees, which include local and national guidelines as well as a diverse range of material readily available via internet resources such as Wikipedia. Modern medicine emphasises evidence-based practice, therefore knowing what information sources shape the knowledge of trainees and how they access this are critical issues. Yet, while there has been some study of what consultant psychiatrists read,^4^ there is a lack of similar work for training grades. We undertook a questionnaire-based survey examining the use of journals and other information sources by psychiatric trainees. We aimed to obtain descriptive data and determine whether patterns of use are related to stage of training.

## Method

### Participants

Questionnaires were distributed at the London Deanery Annual Psychiatry Trainee Conference and the training programmes of a teaching trust in inner London and another in an outer London county.

### Questionnaire

The questionnaire was designed to be easily completed in less than 1 minute. This made it feasible to hand out and collect the questionnaire immediately in person to maximise the response rate, rather than relying on participants' posting or emailing completed questionnaires. A panel of educators and trainees drew up a list of psychiatric and general medical journals from the UK and the USA, on the basis of impact factor or trainee focus. The brevity of the questionnaire required that a small number of representative journals be selected from the large number of possibilities. The text of the questionnaire was written by T.R.W. and then reviewed by R.N. and J.W. to assess clarity and ease of completion.

Questions 1 and 2 ask participants to rate the frequency with which they consult different information sources, including academic journals and online resources, to inform their clinical practice and research. Each item requires the participant to tick a response ranging from ‘never’ to ‘very frequently’ with four intermediate options. Question 3 allows participants a space to optionally list any other information sources they use. The questionnaire also enquires about participants' reasons for consulting journals: question 4 asks to rate the frequency with which trainees consulted journals for each of four specified reasons. Question 5 asks for trainees' aims when consulting academic journals. It was included on the basis that reasons for consulting journals can broadly be separated into two categories: sometimes a trainee may be gathering evidence to answer a specific question but at other times to improve their knowledge generally. The question offers five response options ranging between two extreme options indicating only consulting journals for one of these two reasons and three intermediate options. Question 6 asks participants for their stage of training.

### Analysis

Data from completed questionnaires were coded into a spreadsheet using a scale of 1 (‘never’) to 6 (‘very frequently’) for questions 1, 2 and 4 and a scale of 1 (‘always to answer specific clinical questions’) to 5 (‘always to improve my knowledge generally’) for question 5. Spearman rank correlation coefficients between each of these ordinal responses and year of training (from 1 to 6) were calculated to examine whether advancement in training is associated with use of particular journals and other information sources or particular reasons for consulting them. Significance levels were corrected for multiple testing using the Bonferroni method: raw *P*-values were multiplied by 15 to correct for the 15 correlation coefficients calculated. Data were analysed using the R version 3.02 for Linux (R Foundation for Statistical Computing, Vienna, Austria; www.R-project.org). Free-text responses to question 3 were also entered into the spreadsheet to enable the counting of most common responses.

## Results

**Fig. 1:**
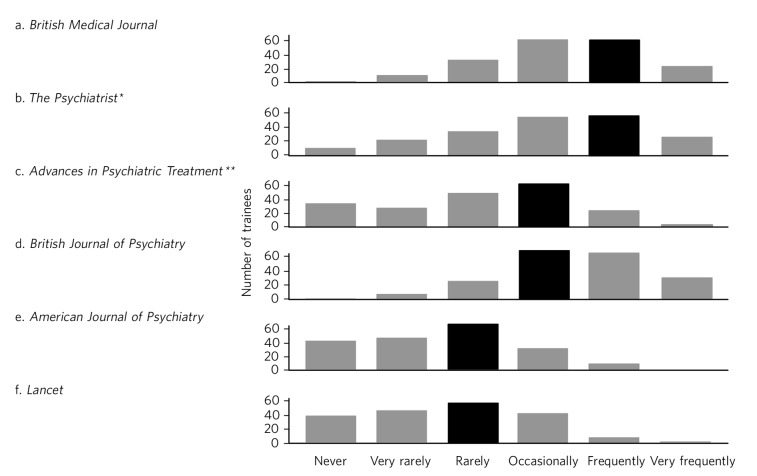
Trainees' reported frequency of consulting different journals. Black bars indicate modal responses. *Now *BJPsych Bulletin*. **Now *BJPsych Advances*.

**Fig. 2:**
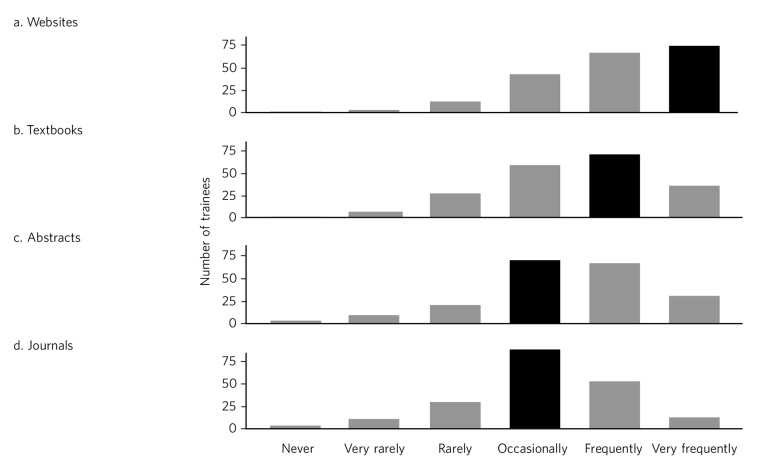
Trainees' reported frequency of using different information sources. Black bars indicate modal responses.

Three hundred questionnaires were distributed and 202 (67%) completed questionnaires returned. One hundred and thirty three of respondents (66%) were core trainees (CT1–3) and the rest were higher trainees (ST4–6). Figures 1 and 2 summarise the frequency with which trainees report consulting different journals and information sources. Spearman's rank correlation coefficients of various outcomes compared with year of training are shown in Table 1. We found relatively small but significant correlations between year of training and sources of information and reasons for retrieving that information.

**Table 1:** Correlations between year of training and questionnaire responses

	Spearman's ρ (d.f.=200)	*P* (Bonferroni^a^)
Information sources^b^		
Academic journals	0.21	0.04
Textbooks	−0.23	0.02
Online abstracts	0.20	n.s.
Websites	−0.09	n.s.

Journals^b^		
*British Journal of Psychiatry*	0.41	<0.001
*BMJ*	0.11	n.s.
*The Psychiatrist* *	0.34	<0.001
*Advances in Psychiatric Treatment* **	0.31	<0.001
*Lancet*	0.04	n.s.
*American Journal of Psychiatry*	0.03	n.s.

Reason for consulting journals^b^		
Help pass exams	0.05	n.s.
Improve clinical practice	0.23	0.01
Inform my own research	0.26	0.004
Interest/enjoyment	0.04	n.s.

Aim when consulting journals^c^		
specific clinical questions *v*. general knowledge	−0.11	n.s.

a. *P*-values have been multiplied by 15 to correct for the multiple comparisons.

b. Correlations between year of training (1 to 6) and questionnaire response coded from 1 ‘never’ to 6 ‘very frequently’.

c. Correlations between year of training (1 to 6) and questionnaire response coded from 1 ‘always to answer specific clinical questions’ to 5 ‘always to improve my knowledge generally’.

n.s., not significant.

* Now *BJPsych Bulletin*.

** Now *BJPsych Advances*.

One hundred and fifteen respondents entered a response to item 3. This section allowed trainees to freely nominate other information sources and demonstrated that senior colleagues (32%), followed by local guidelines (26%), lectures (16%) and National Institute for Health and Care Excellence (NICE) guidelines (10%) were the most frequently used information sources besides journals and websites.

## Discussion

To our knowledge, this is the first study looking at attitudes of psychiatric trainees towards sources of academic information and reading journals. Our questionnaire gathered ratings for the frequency with which trainees read six journals. The pattern of responses may reflect a combination of how useful trainees find the different journals and their availability. Subscription to the *BMJ* is included with membership of the British Medical Association and likewise the *British Journal of Psychiatry*, *BJPsych Bulletin* (formerly *The Psychiatrist*) and *BJPsych Advances* (formerly *Advances in Psychiatric Treatment*) with membership of the Royal College of Psychiatrists. The *American Journal of Psychiatry* and *Lancet* (journals not included with professional memberships commonly held by trainees) are least frequently read. In the case of the *American Journal of Psychiatry* it may also be that trainees seek information most relevant to practice in their own country.

We found several significant correlations between year of training and use of information sources. More advanced trainees consulted journals more frequently and textbooks less frequently. This may be due to advanced trainees requiring knowledge that is more specialised and current than is found in textbooks. It could also reflect higher trainees being more involved in research themselves or having more confidence in reading journals due to the critical appraisal training component of the MRCPsych examinations. More advanced trainees read the *British Journal of Psychiatry*, *BJPsych Bulletin* and *BJPsych Advances* more frequently. This could relate to both the increasing relevance of these journals to staying informed about underpinning science and current clinical practice as a trainee increases in seniority, and their availability: a paper subscription to the *British Journal of Psychiatry* and *BJPsych Bulletin* and online access to *BJPsych Advances* are included with membership of the Royal College of Psychiatrists, a requirement for progression to higher training. There was no significant correlation between training stage and frequency of reading the *BMJ*, the *Lancet* or the *American Journal of Psychiatry*. There was a significant correlation between years of training and how often trainees consulted journals to improve clinical practice or inform their own research. This may reflect more advanced trainees using journals in a way more directed to a specific purpose. We acknowledge that by presenting the data in that format we may miss a different relationship between year of training and frequency of using journals.

Respondents reported consulting websites via internet search engines with greater frequency (modal response ‘very frequently’) than more traditional information sources such as textbooks and journals (modal responses, respectively: ‘frequently’ and ‘occasionally’). Reasons might include the ubiquity of internet access and ease of searching for a specific query. This finding is in line with a study which surveyed specialist physicians, of whom 46% cited internet search engines as a frequent source of information and a further 32% as an occasional source.^5^ A majority of the physicians surveyed reported changing an initial diagnosis on the basis of information from online report tools. There is evidence that using Google to search for terms selected from a patient's case record may assist physicians in making a correct diagnosis and it is plausible that such searching could help develop trainees' skill at differential diagnosis.^6^

### Limitations

Our study has some limitations. Examining in detail which online resources psychiatric trainees are using and for what purpose was beyond the scope of our survey. However, this clearly warrants further research as the frequency with which trainees report using them suggests they may have effects on trainees' education and practice comparable with textbooks and academic journals. Participants were trainees based in and around London and it remains for larger-scale studies to be carried out to see whether our findings generalise to trainees from other parts of the UK.

Our survey shows that in the information age trainees make very frequent use of websites as an information source. Journals are still widely used, especially by more advanced trainees. It is important that trainees continue to be equipped with skills to identify and access high-quality information at the point of clinical uncertainty, and appraise this research rather than rely just on guidelines. Future training curricula and MRCPsych examinations should address and test these skills.
